# Supplementary material for: The Claiming Costs Scale: A new instrument for measuring the costs potential beneficiaries face when claiming social benefits
Source: PLoS One. 2021 Aug 20;16(8):e0256438. doi: 10.1371/journal.pone.0256438 (PMC8378747; doi:10.1371/journal.pone.0256438)
Supplement: S1 Appendix — (DOCX) [file pone.0256438.s001.docx]

**Appendix S1**

**Questionnaire online survey TAKE**

(Sample 1)

This document includes the questions that are used from an online survey for the purpose of writing the article: “The Claiming Costs Scale (CCS): a new instrument for measuring the costs potential beneficiaries face when claiming social benefits.”

*****ENGLISH VERSION*****

1. SCREENING QUESTIONS

[Introduction]. This survey is completely anonymous and confidential. The data collected in this survey will only be used in the context of this study and will not be given to third parties. Participation in this survey is completely voluntary and you can decide to interrupt or stop this survey at any moment.

Please read all the questions carefully before you answer. There are no correct or wrong answers. We are interested in your personal opinion on this topic.

We would like to thank you already for your participation.

A1. Including yourself, how many adult persons are part of your household?

- 1 (1)
- 2 (2)
- 3 (3)
- 4 (4)
- 5 (5)
- 6 (6)
- 7 (7)
- 8 (8)
- 9 (9)
- 10 (10)
- 11 (11)
- 12 (12)
- 13 (13)
- 14 (14)
- 15 (15)
- 16 (16)
- 17 (17)
- 18 (18)
- 19 (19)
- 20 (20)

Display This Question:

If Including yourself, how many adult persons are part of your household? 1 Is Selected

A2_1 The total disposable income of a household consists of: (1) net income from work; (2) social benefits and family allowances; (3) additional income such as rental income, interests, etc.; and (4) income or tips from small jobs, even if these income sources are not declared.

In 2016, was your total disposable household income more or less than 20000 euro ?

- Less than or equal to 20000 euro (1)
- More than 20000 euro (2)

Display This Question:

If Including yourself, how many adult persons are part of your household 1 Is Not Selected

A2_2 The total disposable income of a household consists of: (1) net income from work; (2) social benefits and family allowances; (3) additional income such as rental income, interests, etc.; and (4) income or tips from small jobs, of all household members together even if these income sources are not declared.

In 2016, was your total disposable household income more or less than 35000 euro ?

- Less than or equal to 35000 euro (1)
- More than 35000 euro (2)

A3. In which region do you live?

- Flemish Region (1)
- Walloon Region (2)
- Brussels-Capital Region (3)

1. SOCIO-DEMOGRAPHIC INFORMATION

[Introduction]. In what follows, we will ask you some questions about your socio-demographic situation, your potential experience with applying for benefits, and your overall attitude towards benefits in Belgium. In addition, we will ask you some questions about your current life situation. We will start with some socio-demographic questions.

B1. What is your sex?

- Male (1)
- Female (2)

B2. What is your age?

[open field]

B3. Including yourself, how many adult persons are part of your household?

▼ 1 (1) ... 20 (20)

B4. How many children are part of your household ?

▼ 0 (1) ... 16 (16)

B5. In which province are you living?

- Antwerpen/Anvers (1)
- Limburg/Limbourg (2)
- Oost-Vlaanderen/Frandre orientale (3)
- Vlaams-Brabant /Flemish Brabant (4)
- West-Vlaanderen/ Flandre occidentale (5)
- Henegouwen/Hainaut (6)
- Luik/Liège (7)
- Luxemburg/Luxembourg (8)
- Namen/Namur (9)
- Waals-Brabant/Brabant wallon (10)
- Administratief arrondissement Brussel-Hoofdstad /Quartier administratif de Bruxelles-Capitale (11)

B6. Which description best suits the environment in which you are currently living?

- A large city (1)
- The surburbs of a large city (2)
- A small town (3)
- A village (4)
- The countryside (5)

B7. What is the highest level of education you have obtained? If you completed your education abroad, please give the Belgian equivalent (if recognized in Belgium).

- No degree (1)
- Primary education (2)
- Secondary education (3)
- Higher education (non-university or university) (4)
- Other, please specify: (5) ________________________________________________

B8. What is your main activity status?

- Employed (1)
- Unemployed (2)
- Retired (3)
- Disabled or on sick leave (4)
- Looking after home or family (5)
- Other, please specify (6) ________________________________________________

B9. In general, do you experience problems with understanding Dutch/French?

- Yes, a lot (1)
- Yes, a little (2)
- No, not at all (3)

1. INCOME

[Introduction].The following questions concern the total disposable income of your household. The total disposable income of a household consists of: (1) net income from work; (2) social benefits and family allowances; (3) additional income such as rental income, interests, etc.; and (4) income or tips from small jobs, of all household members together, even if these income sources are not declared.

C1. Please have a look at the following categories. Could you indicate in which category your total monthly disposable household income is situated?

- Less than € 249,99 per month (1)
- € 250,00 - € 499,99 per month (2)
- € 500,00 - € 999,99 per month (3)
- € 1000,00 € - € 1499,99 per month (4)
- € 1500,00 - € 1999,99 per month (5)
- € 2000,00 - € 2499,99 per month (6)
- € 2500,00 - € 2999,99 per month (7)
- € 3000,00 - € 4999,99 per month (8)
- € 5000,00 or more each month (9)

C2. Thinking of your household's total income, is your household able to make ends meet each month, that is pay your usual expenses.....

- Very difficult (1)
- Difficult (2)
- Rather difficult (3)
- Rather easy (4)
- Easy (5)
- Very easy (6)

C3. How much do you think your total disposable household income should be for your household to make ends meet? To answer this question, please take into account your current living conditions and what you understand under “to make ends meet”.

- Monthly amount in euro: (1) ________________________________________________

1. SOCIAL ASSISTANCE FOR PERSONS AT ACTIVE AGE

[Introduction]. Following questions are about the social assistance benefit for persons at active age. This benefit is granted by the Public Centre for Social Welfare (PCSW) to people who have insufficient resources. Previously, the social assistance benefit was also known as the “subsistence minimum”.

D1. Do you know the social assistance benefit?

- Yes (1)
- No (2)

Display This Question:

If Do you know the social assistance benefit? Yes Is Selected

D2. Do you or someone in your household receive a social assistance benefit ?

- Yes (1)
- No (2)

D3. How would you rate your knowledge of the social assistance benefit for persons at active age ? Please rate the following statements by ticking the box that best corresponds with your opinion.

|  | Strongly agree (1) | Agree (2) | Neither agree, nor disagree (3) | Disagree (4) | Strongly disagree (5) |
| --- | --- | --- | --- | --- | --- |
| I know the benefits of the social assistance benefit (1) |  |  |  |  |  |
| I have a fairly good idea whether I am entitled to a social assistance benefit (2) |  |  |  |  |  |
| I find it easy to find all the necessary information on the social assistance benefit (3) |  |  |  |  |  |
| I know the procedure for applying for a social assistance benefit  (4) |  |  |  |  |  |

D4. Do you think the application process for the social assistance benefit is easy or difficult ? Please rate the following statements by ticking the box that best corresponds with your opinion.

|  | Strongly agree (1) | Agree (2) | Neither agree, nor disagree (3) | Disagree (4) | Strongly disagree (5) |
| --- | --- | --- | --- | --- | --- |
| It is a lot of work to apply for a social assistance benefit  (1) |  |  |  |  |  |
| The procedure for applying for a social assistance benefit is difficult (2) |  |  |  |  |  |
| People have to answer a lot of intrusive and personal questions while applying for a social assistance benefit (3) |  |  |  |  |  |
| It is difficult for me to go to the Public Centre for Social Welfare during opening hours to apply for a social assistance benefit (4) |  |  |  |  |  |
| All things considered, it takes a lot of time to claim a social assistance benefit (5) |  |  |  |  |  |

D5. To what extent do you agree or disagree with the following statements about the social assistance benefit ? Please rate the following statements by ticking the box that best corresponds with your opinion.

|  | Strongly agree (1) | Agree (2) | Neither agree, nor disagree (3) | Disagree (4) | Strongly disagree (5) |
| --- | --- | --- | --- | --- | --- |
| It is better that other people do not know if you receive a social assistance benefit (1) |  |  |  |  |  |
| If someone receives a social assistance benefit he or she should be ashamed (2) |  |  |  |  |  |
| People I see regularly, would look down on me if I would receive a social assistance benefit (3) |  |  |  |  |  |
| When I would receive a social assistance benefit, this would give me the feeling that I’m begging (4) |  |  |  |  |  |
| The society is not understanding towards people who are receiving a social assistance benefit (5) |  |  |  |  |  |
| If I would receive a social assistance benefit, I would be ashamed (6) |  |  |  |  |  |

*****DUTCH VERSION*****

1. SCREENING QUESTIONS

[Inleiding]. Deze enquête is volledig anoniem en vertrouwelijk. De gegevens verzameld in deze vragenlijst worden enkel gebruikt voor dit onderzoek en worden niet doorgegeven aan derden. Deelname aan deze enquête is volledig vrijwillig en kan op ieder moment worden stopgezet.

Gelieve alle vragen aandachtig te lezen alvorens u ze beantwoordt. Er zijn zeker geen juiste of foute antwoorden. We zijn hier op zoek naar uw persoonlijke mening over dit onderwerp.

We bedanken u alvast voor uw medewerking!

A1. Hoeveel volwassenen (van 18 jaar of ouder) telt uw huishouden, uzelf inbegrepen?

▼ Click to write Choice 1 (1) ... 20 (20)

Display This Question:

If Hoeveel volwassenen (van 18 jaar of ouder) telt uw huishouden, uzelf inbegrepen? = 1 Is Selected

A2_1 Het **totaal beschikbaar inkomen van een huishouden** bestaat uit, (1) netto inkomsten uit werk, (2) sociale uitkeringen en kinderbijslagen, (3) bijkomende inkomsten zoals huuropbrengsten, intresten, e.a. en (4) vergoedingen om klusjes uit te voeren, of van bijverdiensten (al dan niet aangegeven).

Bedroeg uw totaal beschikbaar huishoudinkomen in 2016 meer of minder dan 20000 euro?

- Minder dan of gelijk aan 20000 euro (1)
- Meer dan 20000 euro (2)

Display This Question:

If Hoeveel volwassenen (van 18 jaar of ouder) telt uw huishouden, uzelf inbegrepen? = 1 Is Not Selected

A2_2 Het **totaal beschikbaar inkomen van een huishouden** bestaat uit, (1) netto inkomsten uit werk, (2) sociale uitkeringen en kinderbijslagen, (3) bijkomende inkomsten zoals huuropbrengsten, intresten, e.a. en (4) vergoedingen om klusjes uit te voeren, of van bijverdiensten, van alle personen uit uw huishouden samen (al dan niet aangegeven).

 Bedroeg uw totaal beschikbaar huishoudinkomen in 2016 meer of minder dan 35000 euro?

- Minder dan of gelijk aan 35000 euro (1)
- Meer dan 35000 euro (2)

A3 In welk gewest bent u woonachtig?

- Vlaams Gewest (1)
- Waals Gewest (2)
- Brussels Hoofdstedelijke Gewest (3)

1. SOCIO-DEMOGRAFISCHE GEGEVENS

[Inleiding]. In wat volgt zullen we u vragen naar een aantal sociale en demografische kenmerken, uw eventuele ervaring met het aanvragen van een uitkering en uw algemene houding ten opzichte van uitkeringen in België. Daarnaast zullen we u enkele korte vragen stellen over uw huidige levenssituatie.

We zullen starten met enkele socio-demografische vragen.

B1. Wat is uw geslacht?

- Man (1)
- Vrouw (2)

B2. Wat is uw leeftijd?

________________________________________________________________

B3. Uit hoeveel personen bestaat uw huishouden, uzelf inbegrepen?

▼ 1 (1) ... 20 (20)

B4. Hoeveel kinderen (jonger dan 18 jaar) telt uw huishouden?

▼ 0 (1) ... 16 (16)

B5. In welke provincie bent u woonachtig?

- Antwerpen (1)
- Limburg (2)
- Oost-Vlaanderen (3)
- Vlaams-Brabant (4)
- West-Vlaanderen (5)
- Henegouwen (6)
- Luik (7)
- Luxemburg (8)
- Namen (9)
- Waals-Brabant (10)
- Administratief arrondissement Brussel-Hoofdstad (11)

B6. Welke omschrijving past het best bij de omgeving waar u woont?

- Een grote stad (1)
- De voorsteden of buitenwijken van een grote stad (2)
- Een kleine stad (3)
- Een dorp (4)
- Op het plattenland (5)

B7. Wat is het hoogste onderwijsdiploma dat u met succes heeft behaald? Indien u uw diploma behaalde in het buitenland, gelieve dan het Belgische equivalent te geven.

- Geen diploma (1)
- Lager onderwijs (2)
- Secundair onderwijs (3)
- Hoger onderwijs (hogeschool of universiteit) (4)
- Andere, specifieer: (5) ________________________________________________

B8. Wat is uw voornaamste activiteit status?

- Werkend (1)
- Werkloos (2)
- Gepensioneerd (3)
- Invalide of arbeidsongeschikt (4)
- Huisman/vrouw (5)
- Andere, specifieer: (6) ________________________________________________

B9. Ervaart u over het algemeen problemen met het begrijpen van het Nederlands?

- Ja, heel erg (1)
- Ja, een beetje (2)
- Nee, helemaal niet (3)

1. INKOMEN

[Inleiding]. De volgende vragen hebben betrekking op het totale inkomen van uw huishouden. **Het totaal beschikbaar inkomen van een huishouden** bestaat uit,
(1) netto inkomsten uit werk,
(2) sociale uitkeringen en kinderbijslagen,
(3) bijkomende inkomens zoals huuropbrengsten, intresten, e.a.
(4) vergoedingen om klusjes uit te voeren, of van bijverdiensten

van alle personen uit uw huishouden samen (al dan niet aangegeven).

C1. Als u de volgende schaal van inkomens bekijkt, kunt u dan zeggen in welke categorie uw totaal beschikbaar huishoudinkomen per maand zich bevindt?

- Minder dan € 249,99 per maand (1)
- € 250,00 - € 499,99 per maand (2)
- € 500,00 - € 999,99 per maand (3)
- € 1000,00 € - € 1499,99 per maand (4)
- € 1500,00 - € 1999,99 per maand (5)
- € 2000,00 - € 2499,99 per maand (6)
- € 2500,00 - € 2999,99 per maand (7)
- € 3000,00 - € 4999,99 per maand (8)
- € 5000,00 of meer per maand (9)

C2. Met het totale beschikbare inkomen van uw huishouden voor ogen, kan uw huishouden zeer gemakkelijk, gemakkelijk, eerder gemakkelijk, eerder moeilijk, moeilijk of zeer moeilijk rond komen.

- Zeer moeilijk (1)
- Moeilijk (2)
- Eerder moeilijk (3)
- Eerder gemakkelijk (4)
- Gemakkelijk (5)
- Zeer gemakkelijk (6)

C3. Hoe groot moet volgens u het totaal beschikbaar inkomen zijn voor uw huishouden om maandelijks juist rond te komen, d.w.z. de eindjes aan elkaar te knopen?
Gelieve voor het beantwoorden van deze vraag rekening te houden met de huidige leefomstandigheden van uw huishouden en met wat u verstaat onder de ‘eindjes aan elkaar knopen’.

- Bedrag per maand in euro (1) ________________________________________________

1. HET LEEFLOON

[Inleiding]. Volgende vragen gaan over **het leefloon**. Dit is een uitkering die door het OCMW wordt gegeven aan mensen die over onvoldoende bestaansmiddelen beschikken. Vroeger stond het leefloon bekend onder de naam "bestaansminimum".

D1. Kent u het leefloon?

- Ja (1)
- Nee (2)

Display This Question:

If Kent u het leefloon? = Ja

D2. Ontvangt u of iemand van uw huishouden een leefloon?

- Ja (1)
- Nee (2)

D3. Hoe zou u uw kennis beoordelen over het aanvragen van een leefloon? Gelieve onderstaande uitspraken te beantwoorden door het bolletje aan te duiden dat het best bij uw mening past.

|  | Helemaal eens (1) | Eens (2) | Niet eens, niet oneens (3) | Oneens (4) | Helemaal oneens (5) |
| --- | --- | --- | --- | --- | --- |
| Ik ken de voordelen van het leefloon (1) |  |  |  |  |  |
| Ik heb een redelijk goed idee of ik in aanmerking kom voor een leefloon (2) |  |  |  |  |  |
| Ik vind het gemakkelijk om alle nodige informatie over het leefloon terug te vinden (3) |  |  |  |  |  |
| Ik ken de procedure voor het aanvragen van een leefloon (4) |  |  |  |  |  |

D4. Hoe eenvoudig of moeilijk denkt u dat de aanvraagprocedure voor een leefloon is? Gelieve volgende uitspraken te beantwoorden door het bolletje aan te duiden dat het best bij uw mening past.

|  | Helemaal eens (1) | Eens (2) | Niet eens, niet oneens (3) | Oneens (4) | Helemaal oneens (5) |
| --- | --- | --- | --- | --- | --- |
| Het is veel werk om het leefloon aan te vragen (1) |  |  |  |  |  |
| De procedure om het leefloon aan te vragen is moeilijk (2) |  |  |  |  |  |
| Mensen moeten veel opdringerige en persoonlijke vragen beantwoorden wanneer ze een leefloon aanvragen (3) |  |  |  |  |  |
| Het is moeilijk voor mij om naar het OCMW kantoor te gaan tijdens de openingsuren om een leefloon aan te vragen (4) |  |  |  |  |  |
| Alles bij elkaar genomen, duurt het lang om het leefloon aan te vragen (5) |  |  |  |  |  |

D5. In welke mate bent u het eens of oneens met volgende stellingen over het leefloon? Gelieve het bolletje aan te duiden dat het best bij uw mening past.

|  | Helemaal eens (1) | Eens (2) | Niet eens, niet oneens (3) | Oneens (4) | Helemaal oneens (5) |
| --- | --- | --- | --- | --- | --- |
| Het is beter dat andere personen niet weten dat je een leefloon ontvangt (1) |  |  |  |  |  |
| Als iemand het leefloon krijgt, moet die zich daar voor schamen (2) |  |  |  |  |  |
| Mensen in mijn omgeving kijken op me neer als ik een leefloon zou krijgen (3) |  |  |  |  |  |
| Wanneer ik een leefloon zou ontvangen, zou dit me het gevoel geven dat ik bedel (4) |  |  |  |  |  |
| De maatschappij is onbegripvol ten opzichte van mensen die een leefloon ontvangen (5) |  |  |  |  |  |
| Als ik een leefloon zou krijgen, dan zou ik me daar voor schamen (6) |  |  |  |  |  |

*****FRENCH VERSION*****

1. SCREENING QUESTIONS

[Introduction] Cette étude est totalement anonyme et confidentielle. Les données ne seront utilisées que pour cette recherche et ne seront pas divulguées à des tiers. La participation à cette étude est entièrement volontaire et peut être interrompue à tout moment.

Veuillez lire attentivement toutes les questions avant d’y répondre. Il n'y a pas de bonne ni de mauvaise réponse, nous sommes intéressés par votre opinion sur le sujet traité.

Nous vous remercions d’avance de votre coopération!

A1. En vous comptant, combien d’adultes (de 18 ans ou plus) font partie de votre ménage?

▼ Click to write Choice 1 (1) ... 20 (20)

Display This Question:

If En vous comptant, combien d’adultes (de 18 ans ou plus) font partie de votre ménage? = 1 Is Selected

A2_1 **Le revenu disponible total d’un ménage** se compose (1) des salaires nets, traitements et revenus professionnels nets des indépendants, (2) des prestations sociales et allocations familiales, (3) des revenus complémentaires tels que recettes de location, intérêts, etc. (4) des revenus de petits travaux ou honoraires/pourboires (déclaré ou non).

 En 2016, le revenu total disponible de votre ménage était-il supérieur ou inférieur à 20000 euros?

- Moins de ou égal à 20000 euros (1)
- Plus de 20000 euros (2)

Display This Question:

If En vous comptant, combien d’adultes (de 18 ans ou plus) font partie de votre ménage? = 1 Is Not Selected

A2_2 **Le revenu disponible total d’un ménage** se compose (1) des salaires nets, traitements et revenus professionnels nets des indépendants, (2) des prestations sociales et allocations familiales, (3) des revenus complémentaires tels que recettes de location, intérêts, etc. (4) des revenus de petits travaux ou honoraires/pourboires des différents membres du ménage pris ensemble (déclaré ou non).

 En 2016, le revenu total disponible de votre ménage était-il supérieur ou inférieur à 35000 euros?

- Moins de ou égal à 35000 euros (1)
- Plus de 35000 euros (2)

Dans quelle Région habitez-vous?

- La Région flamande (1)
- La Région wallonne (2)
- La Région Bruxelles-Capitale (3)

1. DONEES SOCIO-DEMOGRAPHIQUES

[Introduction]. Dans ce qui suit, nous vous poserons des questions sur vos caractéristiques socio-démographiques, sur votre expérience éventuelle lors d’une procédure de demande d’allocation et sur votre attitude générale à l'égard des allocations en Belgique. Nous vous poserons également quelques petites questions sur votre situation actuelle.

Nous commencerons par quelques questions socio-démographiques.

B1. Quel est votre sexe?

- Masculin (1)
- Féminin (2)

B2. Quel est votre âge?

________________________________________________________________

B3. En vous incluant, combien de personnes compte votre ménage?

▼ Click to write Choice 1 (1) ... 20 (20)

B4. Combien d’enfants (de moins de 18 ans) compte votre ménage?

▼ 0 (1) ... 16 (17)

B5. Dans quelle Province vivez-vous?

- Anvers (1)
- Limbourg (2)
- Flandre orientale (3)
- Brabant flamand (4)
- Flandre occidentale (5)
- Hainaut (6)
- Liège (7)
- Luxembourg (8)
- Namur (9)
- Brabant wallon (10)
- Une commune de Bruxelles-Capitale (11)

B6. Quelle description convient le mieux à l'environnement dans lequel vous vivez?

- Une grande ville (1)
- La banlieue ou la banlieue d’une grande ville (2)
- Une petite ville (3)
- Un village (4)
- À la campagne (5)

| Page Break |  |
| --- | --- |

B7. Quel est le plus haut diplôme d'études que vous ayez obtenu? Si vous avez étudié à l'étranger, veuillez donner l'équivalence belge.

- Aucun diplôme (1)
- Certificat d'études primaires (CEB) (2)
- Enseignement secondaire (3)
- Enseignement supérieur (non-universitaire ou universitaire) (4)
- Autre, précisez: (5) ________________________________________________

B8. Quel est votre statut principal d'activité?

- Actif professionnellement (1)
- Sans emploi (2)
- Retraité (3)
- Invalide ou en arrêt maladie (4)
- Au foyer (5)
- Autre, précisez: (6) ________________________________________________

B9. Da manière générale, avez-vous des difficultés pour comprendre le français?

- Oui, beaucoup (1)
- Oui, un peu (2)
- Non, pas du tout (3)

1. REVENU

[Introduction]. Les questions suivantes concernent **le revenu total disponible de votre ménage**. Le revenu disponible total d’un ménage se compose
 (1) des salaires nets, traitements et revenus professionnels nets des indépendants,
 (2) des prestations sociales et allocations familiales,
 (3) des revenus complémentaires tels que recettes de location, intérêts, etc.
 (4) des revenus de petits travaux ou honoraires /pourboires

des différents membres du ménage pris ensemble (déclarés ou non).

C1. Merci de regarder les catégories suivantes et de m’indiquer dans quelle tranche se trouve le revenu moyen mensuel de votre ménage (en incluant salaires, prestations sociales, recettes de location, pourboires,…).

- Moins de € 249,99 par mois (1)
- € 250,00 - € 499,99 par mois (2)
- € 500,00 - € 999,99 par mois (3)
- € 1000,00 € - € 1499,99 par mois (4)
- € 1500,00 - € 1999,99 par mois (5)
- € 2000,00 - € 2499,99 par mois (6)
- € 2500,00 - € 2999,99 par mois (7)
- € 3000,00 - € 4999,99 par mois (8)
- € 5000,00 ou plus par mois (9)

C2. Pensez aux revenus totaux de votre ménage (par semaine ou par mois). Votre ménage est-il en mesure de joindre les deux bouts, c’est-à-dire de payer vos dépenses habituelles?

- Très difficilement (1)
- Difficilement (2)
- Plutôt difficilement (3)
- Plutôt facilement (4)
- Facilement (5)
- Très facilement (6)

C3. Quel est le montant du revenu total disponible (après déduction de l'impôt sur le revenu des personnes physiques et des cotisations de sécurité sociale) dont votre ménage devrait disposer mensuellement pour s’en sortir, pour joindre les deux bouts ?
 Veuillez tenir compte des conditions de vie actuelle de votre ménage et de ce que vous entendez par « joindre les deux bouts » pour répondre à cette question.

- Montant par mois en euros (1) ________________________________________________

1. LE REVENU D’INTEGRATION SOCIALE

D1. Les questions suivantes concernent **le revenu d’intégration sociale**. Cette allocation est accordée par le CPAS aux personnes qui n'ont pas suffisamment de ressources. Auparavant, le salaire décent était connu sous le nom de «minimex» (minimum de moyens d’existence).

Q112 Connaissez-vous le revenu d'intégration sociale?

- Oui (1)
- Non (2)

End of Block

Display This Question:

If Connaissez-vous le revenu d'intégration sociale?= Oui

Q84 Vous ou un autre membre de votre ménage reçoit-il le revenu d'intégration sociale?

- Oui (1)
- Non (2)

D3. Comment évalueriez-vous vos connaissances de la procédure de demande du revenu d’intégration sociale? Veuillez évaluer les propositions suivantes en indiquant la réponse qui correspond à votre opinion.

|  | Tout à fait d’accord (1) | D’accord (2) | Ni d'accord, ni pas d'accord (3) | Pas d’accord (4) | Pas du tout d’accord (5) |
| --- | --- | --- | --- | --- | --- |
| Je connais les avantages du revenu d'intégration sociale (1) |  |  |  |  |  |
| Je sais assez bien si j'ai droit au revenu d'intégration sociale ou non (2) |  |  |  |  |  |
| Je pense qu'il est facile de trouver les informations nécessaires sur le revenu d'intégration sociale (3) |  |  |  |  |  |
| Je connais la procédure de demande du revenu d'intégration sociale (4) |  |  |  |  |  |

D4. La procédure de demande du revenu d'intégration sociale est-il facile ou difficile? Veuillez évaluer les propositions suivantes en indiquant la réponse qui correspond à votre opinion.

|  | Tout à fait d’accord (1) | D’accord (2) | Ni d'accord, ni pas d'accord (3) | Pas d’accord (4) | Pas du tout d’accord (5) |
| --- | --- | --- | --- | --- | --- |
| C'est beaucoup de travail de demander le revenu d'intégration sociale (1) |  |  |  |  |  |
| La procédure de demande du revenu d'intégration sociale est compliquée (2) |  |  |  |  |  |
| Les personnes doivent répondre à de nombreuses questions intrusives et personnelles lorsqu'elles demandent le revenu d'intégration sociale (3) |  |  |  |  |  |
| Il est difficile d'aller au CPAS durant les heures d'ouverture pour faire une demande de revenu d'intégration sociale (4) |  |  |  |  |  |
| En tenant compte de tout, la demande de revenu d'intégration sociale prend beaucoup de temps (5) |  |  |  |  |  |

D5. A quel point êtes-vous d'accord ou pas d'accord avec les déclarations suivantes concernant le revenu d'intégration sociale? Veuillez évaluer les propositions suivantes en indiquant la réponse qui correspond à votre opinion.

|  | Tout à fait d’accord (1) | D’accord (2) | Ni d'accord, ni pas d'accord (3) | Pas d’accord (4) | Pas du tout d’accord (5) |
| --- | --- | --- | --- | --- | --- |
| Il est préférable que les autres personnes ne sachent pas que vous bénéficiez du revenu d'intégration sociale (1) |  |  |  |  |  |
| Si quelqu’un obtient le revenu d’intégration sociale, il doit avoir honte (2) |  |  |  |  |  |
| Les personnes que je fréquente me prendraient de haut si je recevais le revenu d’intégration sociale (3) |  |  |  |  |  |
| Recevoir le revenu d'intégration sociale me donnerait le sentiment de mendier (4) |  |  |  |  |  |
| En général, l'opinion publique n'est pas compréhensif vis-à-vis des personnes bénéficiaires du revenu d'intégration sociale (5) |  |  |  |  |  |
| Si j'avais un revenu d'intégration sociale, j’en aurais honte (6) |  |  |  |  |  |
