# Supplementary material for: The Claiming Costs Scale: A new instrument for measuring the costs potential beneficiaries face when claiming social benefits
Source: PLoS One. 2021 Aug 20;16(8):e0256438. doi: 10.1371/journal.pone.0256438 (PMC8378747; doi:10.1371/journal.pone.0256438)
Supplement: S2 Appendix — (DOCX) [file pone.0256438.s002.docx]

**Appendix S2**

**Questionnaire TAKE Survey**

**Measuring the non-take-up of public provisions among low-income families in Belgium.**

(Sample 2)

*****ENGLISH VERSION*****

This document includes the questions that are used from the TAKE Survey for the purpose of writing the article: “The Claiming Costs Scale (CCS): a new instrument for measuring the costs potential beneficiaries face when claiming social benefits.”

The TAKE survey was conducted by professional and trained interviewers and consisted of a face-to-face interview with the household head (main interview) and additional, shorter interviews with the other adult household members. The questions below are an extract of the main questionnaire.

1. INTRODUCTION, INFORMED CONSENT AND SELECTION MAIN RESPONDENT

Q752 **Please, encode the household ID.**

________________________________________________________________

Q836 **[IWER: please select the Region where the household lives.]**

- Brussels (1)
- Flanders (2)
- Wallonia (3)

QID80 THIS MODULE IS ASKED ONLY ONCE TO THE REFERENCE PERSON. PLEASE SELECT IT WITH CAUTION.

QID81 [GENERAL INTRODUCTION INTERVIEWER]

**In this study, we aim to investigate how accessible social benefits and services are in Belgium and how many people use or don’t use them. In particular, we want to investigate whether there are many people who are entitled to a benefit, but do not receive it.**

**To this end, I will ask you questions about your family composition, your housing situation, your potential employment, your experience with applying for benefits, or about the reasons why you did not apply for benefits in the past, etc. I will also ask you some financial questions. These questions are needed to determine whether or not a person is entitled to social assistance. If we come to a question which you do not want to answer, just let me know and I will go on to the next question.**

**I can guarantee that all your answers will be treated anonymously and confidentially. Your participation is completely voluntary and whether you answer to these questions or not, the benefits to which you are entiteld will remain in any case guaranteed.**

Q754 [INFORMED CONSENT]
**To keep the interview as short as possible, we would like to link your answers with some data already existing in administrative databases. It concerns: the right to social assistance and information on entitlement criteria. In order to avoid having to ask you this information again, I would like to ask your permission that the researchers of this study are allowed to use the already existing administrative data.

Both these data and your answers to the questionnaire will only be used by the researchers involved in this study. All data is processed 'encrypted', with full respect for the privacy legislation. It means that your name and address remain anonymous. In addition, the collected data will only be used for statistical purposes and in no case for administrative purposes.

Finally, I would like to emphasize again that participating in this study and agreeing with the use of your administrative data will not cause any negative consequences and will certainly not affect your current rights.**
 
**Do you agree that we link your answers with administrative data?**

- Yes (1)
- No [IWER: the interview immediately stops.] (2)

Skip To: End of Survey If [Interviewer's introduction about administrative datalinkage]To keep the interview as short as po... = No [IWER: the interview immediately stops.]

|  |
| --- |

QID82 [IDENTIFICATION OF THE REFERENCE PERSON]

**Are you the person within your household who is mainly responsible for applying for potential benefits and has good knowledge of the household finances? If you are not this person, then could you tell me who is?**

- Yes, the respondent has enough knowledge to answer this questionnaire (1)
- No, someone else in the household is better informed to participate in this interview. [IWER: the interview immediately stops.] (2)

Skip To: End of Survey If Are you the person within your household who is mainly responsible for applying for potential benefits and has good knowledge of the household finances? If you are not this person, then could you tell me who is? = No, someone else in the household is better informed to fill in this questionnaire. [IWER: the interview immediately stops.]

Q848 **[IWER: Write down the respondent's gender according to your observation. Ask in doubt.]**

- Man (1)
- Woman (2)
- Transgender (3)

Q584
**How old are you?**

 [IWER: In case the respondent does not know the age, please encode 200.]

________________________________________________________________

1. HOUSEHOLD COMPOSITION

QID85 **Let's start with the composition of your household and the relationships you have with your household members.**

QID86 **NOT INCLUDING YOURSELF, how many people - including children - currently live here regularly as members of this household? A household consists of all the persons who live at the SAME ADDRESS (have the same front door) and who have a COMMON HOUSEHOLD BUDGET or USUALLY EAT TOGETHER.**

[IWER: The following persons may be members of the household: children living with their parents on the same address, or parents who live with their children on the address of the children; children who live part-time with the respondent, even when they are legally registered as living with the ex-partner (or elsewhere), but are financially dependent from the respondent; persons officially registered as living on this address and in case of financial dependency, but who temporarily work or study too far away to commute every day (e.g.'kotstudenten' / 'kots d'étudiant'); persons who reside temporarily in a hospital, foreign country or elsewhere, if the respondent considers them to be household members.
Usually not considered household members: tenants (renting a room), even when they sometimes eat together with the respondent, unless the respondent explicitly mentions them as household members.]

________________________________________________________________

Skip To: End of Block If Condition: NOT INCLUDING YOURSELF, how many people - including children - currently live here regularly as members of this household? A household consists of all the persons who live at the SAME ADDRESS (have the same front door) and who have a COMMON HOUSEHOLD BUDGET or USUALLY EAT TOGETHER. [IWER: The following persons may be members of the household: children living with their parents on the same address, or parents who live with their children on the address o ... Is Equal to 0. Skip To: End of Block.

|  |
| --- |

Q341 **STILL NOT INCLUDING YOURSELF, how many of them are adult members (18 years old and more) of the household?**

________________________________________________________________

Q343 **[Iwer: Please, simply enter the following number of non-adult members]** $e{ q://QID86/ChoiceTextEntryValue - q://QID342/ChoiceTextEntryValue }

________________________________________________________________

1. SOCIO-DEMOGRAPHIC INFORMATION

Q154 [INTRODUCTION]

**From here, we will start with the individual part of the questionnaire. I would like to start this part with asking you some questions about your PERSONAL situation. We will start with some questions about your nationality, education and potential job(s).**

Q161 **What is the highest level of education you have obtained?**
[IWER: Spontaneous response. Encode the correct option and confirm loudly with the respondent.]
[IWER: Only one answer possible.]

- 1. Primary education (1)
- 2. Lower secondary education (including general, artistic, technical or vocational education) (2)
- 3. Upper secondary education (including general, artistic, technical or vocational education) (3)
- 4. Non-university higher education of short cyclus (2 or 3 years) (4)
- 5. Non-university higher education of long cyclus (4 or 5 years) (5)
- 6. University education (6)
- 7. None (7)
- 8. Other but not recognized in Belgium (8)
- 9. Other, please specify: (9) ________________________________________________
- Don’t know (10)
- Refusal (11)

Q162 **Please, look at showcard 2. Which of the following describes your main current work status best?**
[IWER: Only one possible answer.]

- 1. Working (in salary) (1)
- 2. Working (self-employed) (2)
- 3. Unemployed (3)
- 4. Employment program (for example article 60) (4)
- 5. In vocational training/re-education/education (5)
- 6. Parental leave (6)
- 7. In retirement or early retirement (7)
- 8. Unemployment with increased company supplement (aka bridge pension; for older workers who have been fired) (8)
- 9. Permanently sick or disabled (9)
- 10. Looking after home or family (10)
- 11. Career break or ‘time credit’ and registered with RVA/ ONEM (11)
- 12. Other, please specify: (12) ________________________________________________
- Don’t know (13)
- Refusal (14)

1. HOUSEHOLD INCOME

Q135 **The following questions concern the total income of YOUR HOUSEHOLD. The total available income of a household consists of: (1) net incomes from work;** **(2) social security benefits (for example the social assistance benefit), family allowances, etc;** **(3) additional income such as rental income, etc. and;** **(4) income from small jobs or fees / tips from ALL household members taken TOGETHER, even if not declared.**

**In case you are in a collective debt counselling, to calculate your total household income, please take into account the amount of your income that monthly goes to the repayment of your debts.**
**In case you have to pay alimony, to calculate your total household income, please take into account the amount of alimony you have to pay each month. In case you receive alimony, please do not take into account the amount you receive each month to answer this question.**

Q136 **How much was your monthly total disposable HOUSEHOLD income last month? If this income varies from month to month, could you please give an average?**

- Amount per month: (1) ________________________________________________
- Don’t know (2)
- Refusal (3)

Display This Question:

If How much was your monthly total disposable HOUSEHOLD income last month? If this income varies from month to month, could you please give an average? = Don’t know

Or How much was your monthly total disposable HOUSEHOLD income last month? If this income varies from month to month, could you please give an average? = Refusal

Q137 **Please look at showcard 10. Could you tell me in which category your total, monthly available HOUSEHOLD income (inclusive wages, benefits, rental income, extra earnings, etc.) from last month is situated?**

- 1. Less than 249€ per month (1)
- 2. Between 250 and 499€ per month (2)
- 3. Between 500 and 749€ per month (3)
- 4. Between 750 and 999€ per month (4)
- 5. Between 1000 and 1499€ per month (5)
- 6. Between 1500 and 1999€ per month (6)
- 7. Between 2000 and 2499€ per month (7)
- 8. Between 2500 and 2999€ per month (8)
- 9. Between 3000 and 4999€ per month (9)
- 10. 5000€ or more per month (10)
- Don’t know (11)
- Refusal (12)

Q140 **Thinking of your household's total income, is your household able to make ends meet each month, that is pay your usual expenses.....**

[IWER: Read out options below.]

- 1. With great difficulty (1)
- 2. With difficulty (2)
- 3. With some difficulty (3)
- 4. Fairly easily (4)
- 5. Easily (5)
- 6. Very easily (6)
- Don’t know (7)
- Refusal (8)

1. SOCIAL ASSISTANCE FOR PERSONS AT ACTIVE AGE

Q229 **Do you know the social assistance benefit for persons at active age?**

- Yes (1)
- No (2)
- Don’t know (3)
- Refusal (4)

Display This Question:

If Do you know the social assistance benefit for persons at active age?  = No

Or Do you know the social assistance benefit for persons at active age?  = Don’t know

Or Do you know the social assistance benefit for persons at active age?  = Refusal

Q230 [IWER: read the following description.]

**The social assistance benefit is a type of financial assistance granted to people who have difficulties with making ends meet. The social assistance benefit is part of the right to social integration, which was formerly called the subsistence minimum income. In order to receive a social assistance benefit you must meet a number of entitlement conditions and apply for it at a local PCSW office in your neighborhood.**

**Do you recognize this benefit after hearing the description?**

- Yes (1)
- No (2)
- Don’t know (3)
- Refusal (4)

Skip To: End of Block If [IWER: read the following description.]   The social assistance benefit is a type of financial assistance granted to people who have difficulties with making ends meet. The social assistance benefit is part of the right to social integration, which was formerly called the subsistence minimum income. In order to receive a social assistance benefit you must meet a number of entitlement conditions and apply for it at a local PCSW office in your neighborhood. Do you recognize this benefit after hearing the description? = No

Skip To: End of Block If [IWER: read the following description.]   The social assistance benefit is a type of financial assistance granted to people who have difficulties with making ends meet. The social assistance benefit is part of the right to social integration, which was formerly called the subsistence minimum income. In order to receive a social assistance benefit you must meet a number of entitlement conditions and apply for it at a local PCSW office in your neighborhood. Do you recognize this benefit after hearing the description? = Don’t know

Skip To: End of Block If [IWER: read the following description.]   The social assistance benefit is a type of financial assistance granted to people who have difficulties with making ends meet. The social assistance benefit is part of the right to social integration, which was formerly called the subsistence minimum income. In order to receive a social assistance benefit you must meet a number of entitlement conditions and apply for it at a local PCSW office in your neighborhood. Do you recognize this benefit after hearing the description? = Refusal

Q244 **Please, look at showcard 15. I will read you some statements about your knowledge of applying for a social assistance benefit for persons at active age. For each statement, could you tell me if you agree or not.**

**I know the benefits of the social assistance benefit.**

- 1. Strongly agree (1)
- 2. Agree (2)
- 3. Neither agree, nor disagree (3)
- 4. Disagree (4)
- 5. Strongly disagree (5)
- Don’t know (6)
- Refusal (7)

Q444 **(Please look at showcard 15.)**

**I have a fairly good idea whether I am entitled to a social assistance benefit.**

- 1. Strongly agree (1)
- 2. Agree (2)
- 3. Neither agree, nor disagree (3)
- 4. Disagree (4)
- 5. Strongly disagree (5)
- Don’t know (6)
- Refusal (7)

Q445 **(Please look at showcard 15.)**

**I know the procedure for applying for a social assistance benefit.**

- 1. Strongly agree (1)
- 2. Agree (2)
- 3. Neither agree, nor disagree (3)
- 4. Disagree (4)
- 5. Strongly disagree (5)
- Don’t know (6)
- Refusal (7)

Q245 **(Please look at showcard 15) I will read you some statements about the application procedure for the social assistance benefit. For each one, could you tell me if you agree or not.**

**It is a lot of work to apply for a social assistance benefit.**

- 1. Strongly agree (1)
- 2. Agree (2)
- 3. Neither agree, nor disagree (3)
- 4. Disagree (4)
- 5. Strongly disagree (5)
- Don’t know (6)
- Refusal (7)

Q570 **(Please look at showcard 15.)**

**The procedure for applying a social assistance benefit is difficult.**

- 1. Strongly agree (1)
- 2. Agree (2)
- 3. Neither agree, nor disagree (3)
- 4. Disagree (4)
- 5. Strongly disagree (5)
- Don’t know (6)
- Refusal (7)

Q567 **(Please look at showcard 15.)**

**All things considered, it takes a lot of time to claim a social assistance benefit.**

- 1. Strongly agree (1)
- 2. Agree (2)
- 3. Neither agree, nor disagree (3)
- 4. Disagree (4)
- 5. Strongly disagree (5)
- Don’t know (6)
- Refusal (7)

Q460 **Please, look at showcard 15. I will read you some statements about the social assistance benefit. For each one could you tell me if you agree or not?**

**If someone receives a social assistance benefit, he or she should feel ashamed.**

- 1. Strongly agree (1)
- 2. Agree (2)
- 3. Neither agree, nor disagree (3)
- 4. Disagree (4)
- 5. Strongly disagree (5)
- Don’t know (6)
- Refusal (7)

Q459 **(Please look at showcard 15.)**

**People I see regularly would look down on me if I would receive a social assistance benefit.**

- 1. Strongly agree (1)
- 2. Agree (2)
- 3. Neither agree, nor disagree (3)
- 4. Disagree (4)
- 5. Strongly disagree (5)
- Don’t know (6)
- Refusal (7)

Q456 **(Please look at showcard 15.) When I would receive a social assistance benefit, this would give me the feeling that I’m begging.**

- 1. Strongly agree (1)
- 2. Agree (2)
- 3. Neither agree, nor disagree (3)
- 4. Disagree (4)
- 5. Strongly disagree (5)
- Don’t know (6)
- Refusal (7)

Q461 **(Please look at showcard 15.)**

**If I would receive a social assistance benefit, I would feel ashamed.**

- 1. Strongly agree (1)
- 2. Agree (2)
- 3. Neither agree, nor disagree (3)
- 4. Disagree (4)
- 5. Strongly disagree (5)
- Don’t know (6)
- Refusal (7)

*****DUTCH VERSION*****

1. INTRODUCTION, INFORMED CONSENT AND SELECTION MAIN RESPONDENT

Q752 **Gelieve het huishoud ID in te geven.**

________________________________________________________________

Q836 [IWER: **Gelieve het gewest aan te duiden waar het huishouden woonachtig is**.]

- Brussels Hoofdstedelijk Gewest (1)
- Vlaams Gewest (2)
- Waals Gewest (3)

QID80 Deze module is enkel voor de referentiepersoon. Gelieve deze zorgvuldig te selecteren.

QID81 [Algemene inleiding interviewer]

**In deze studie willen we onderzoeken hoe toegankelijk sociale uitkeringen en diensten in België zijn en hoeveel mensen er wel of niet gebruik van maken. In het bijzonder willen we nagaan of er veel mensen zijn die wel recht hebben op een uitkering, maar deze niet krijgen. Om die reden, zal ik u vragen stellen over uw gezinssamenstelling, uw woonsituatie, uw eventuele tewerkstelling, uw ervaring met het aanvragen van uitkeringen, of redenen waarom u dit in het verleden niet heeft gedaan, enz. Ik zal u ook een aantal financiële vragen stellen. Deze zijn nodig om vast te stellen of iemand recht heeft op sociale uitkeringen en kortingen. Als we aan een vraag komen die u niet wilt beantwoorden, laat u me dat dan gewoon weten en ik ga verder met de volgende vraag. Ik garandeer u dat al uw antwoorden op deze verschillende modules anoniem en vertrouwelijk behandeld zullen worden. Uw deelname is volledig vrijwillig en of u deze vragen nu beantwoordt of niet, de tegemoetkomingen waar u recht op heeft zullen in elk geval gegarandeerd blijven.**

Q754 [Inleiding door interviewer aangaande administratieve datakoppeling]
**Om het interview zo kort mogelijk te houden, zouden we graag uw antwoorden koppelen aan reeds bestaande gegevens uit administratieve bronnen, namelijk over (1) het recht op sociale uitkeringen of kostenverminderingen en (2) kenmerken die bepalend zijn voor het al dan niet in aanmerking komen voor uitkeringen. Om te vermijden dat ik u opnieuw naar deze informatie moet vragen, zou ik uw toestemming willen vragen opdat de onderzoekers de reeds bestaande administratieve gegevens mogen gebruiken.

Zowel deze gegevens als de antwoorden op de vragenlijst zullen enkel worden gebruikt door de onderzoekers. Alle gegevens worden gecodeerd verwerkt, met volledig respect voor de privacywetgeving, i.e. uw naam en adres blijven anoniem. Daarnaast zullen de verzamelde gegevens slechts voor statistische doeleinden gebruikt worden en in geen geval voor administratieve doeleinden.

Als laatste wil ik nog eens benadrukken dat een deelname aan deze studie en het akkoord gaan met het gebruiken van uw administratieve data in geen geval negatieve gevolgen met zich zal meebrengen en op geen enkele manier invloed zal hebben op uw huidige rechten.**
 
**Gaat u ermee akkoord dat we u antwoorden linken aan administratieve data?**

- Ja (1)
- Nee [IWER: de vragenlijst eindigt hier] (2)

Skip To: End of Survey If [Inleiding door interviewer aangaande administratieve datakoppeling]Om het interview zo kort mogelijk te houden, zouden we graag uw antwoorden koppelen aan reeds bestaande gegevens uit administratieve bronnen, namelijk over (1) het recht op sociale uitkeringen of kostenverminderingen en (2) kenmerken die bepalend zijn voor het al dan niet in aanmerking komen voor uitkeringen. Om te vermijden dat ik u opnieuw naar deze informatie moet vragen, zou ik uw toestemming willen vragen opdat de onderzoekers de reeds bestaande administratieve gegevens mogen gebruiken. Zowel deze gegevens als de antwoorden op de vragenlijst zullen enkel worden gebruikt door de onderzoekers. Alle gegevens worden gecodeerd verwerkt, met volledig respect voor de privacywetgeving, i.e. uw naam en adres blijven anoniem. Daarnaast zullen de verzamelde gegevens slechts voor statistische doeleinden gebruikt worden en in geen geval voor administratieve doeleinden. Als laatste wil ik nog eens benadrukken dat een deelname aan deze studie en het akkoord gaan met het gebruiken van uw administratieve data in geen geval negatieve gevolgen met zich zal meebrengen en op geen enkele manier invloed zal hebben op uw huidige rechten.   Gaat u ermee akkoord dat we u antwoorden linken aan administratieve data? = Nee [IWER: de vragenlijst eindigt hier]

QID82 [IDENTIFICATIE VAN DE REFERENTIEPERSOON]

**Bent u de persoon binnen uw huishouden die hoofdzakelijk verantwoordelijk is voor het aanvragen van eventuele uitkeringen en goed op de hoogte is van de financiën van het huishouden? Indien u niet deze persoon bent, kunt u me dan vertellen wie in dit huishouden dit wel is?**

- Ja, de respondent zelf is voldoende op de hoogte om deze vragenlijst te beantwoorden (1)
- Nee, iemand anders binnen dit huishouden is beter geplaatst deze vragenlijst te beantwoorden, specifieer wie [IWER: mondeling antwoord, interview stopt met huidige respondent en nieuw interview start met gesuggereerd persoon] (2)

Skip To: End of Survey If Bent u de persoon binnen uw huishouden die hoofdzakelijk verantwoordelijk is voor het aanvragen van eventuele uitkeringen en goed op de hoogte is van de financiën van het huishouden? Indien u niet deze persoon bent, kunt u me dan vertellen wie in dit huishouden dit wel is? = Nee, iemand anders binnen dit huishouden is beter geplaatst deze vragenlijst te beantwoorden, specifieer wie [IWER: mondeling antwoord, interview stopt met huidige respondent en nieuw interview start met gesuggereerd persoon]

Q849 [IWER: Duidt het geslacht van de respondent aan op basis van observatie. Stel deze vraag enkel luidop indien u twijfelt.]

- Man (1)
- Vrouw (2)
- Transgender (3)

Q584
**Hoe oud bent u?**

 [IWER: Indien de respondent de leeftijd niet kent, gelieve dan de code 200 te noteren.]

________________________________________________________________

1. HOUSEHOLD COMPOSITION

QID85 **Laten we beginnen met enkele vragen over uw huishoudsamenstelling en de relaties die u heeft met uw huishoudleden.**

QID86 **UZELF ER NIET BIJGETELD, hoeveel personen - inclusief kinderen - wonen hier gewoonlijk als leden van dit huishouden? Een huishouden bestaat uit alle personen die op HETZELFDE ADRES wonen (dezelfde voordeur hebben) en een GEMEENSCHAPPELIJK huishoudbudget hebben of meestal samen eten.**

[IWER: De volgende personen kunnen leden van het huishouden zijn: kinderen die met hun ouders op hetzelfde adres wonen, of ouders die met hun kinderen op het adres van de kinderen wonen; kinderen die deeltijds bij de respondent wonen en deeltijds bij de ex-partner, zelfs al staan ze wettelijk ingeschreven op het adres van de ex-partner (of ergens anders), maar financieel afhankelijk zijn van de respondent; personen die officieel staan ingeschreven op dit adres en financieel afhankelijk zijn van de respondent, maar die tijdelijk werken of studeren op een andere plek die te ver van huis is om elke dag van en naar te pendelen (bijvoordbeeld 'kotstudenten'); personen die tijdelijk in het ziekenhuis, het buitenland of ergens anders verblijven, indien de respondent deze ook als huishoudleden beschouwt.
Personen die meestal niet als leden van het huishouden worden beschouwd: inwonende huurders (die bijvoorbeeld een kamer huren), zelfs al eten ze af en toe mee met de respondent, tenzij de respondent deze uitdrukkelijk als leden van het huishouden beschouwd.]

________________________________________________________________

Skip To: End of Block If Condition: UZELF ER NIET BIJGETELD, hoeveel personen - inclusief kinderen - wonen hier gewoonlijk als leden van dit huishouden? Een huishouden bestaat uit alle personen die op HETZELFDE ADRES wonen (dezelfde voordeur hebben) en een GEMEENSCHAPPELIJK huishoudbudget hebben of meestal samen eten. [IWER: De volgende personen kunnen leden van het huishouden zijn: kinderen die met hun ouders op hetzelfde adres wonen, of ouders die met hun kinderen op het adres van de kinderen wonen; kin ... Is Equal to 0. Skip To: End of Block.

Q341 **UZELF ER NOG STEEDS NIET BIJGETELD, hoeveel van hen zijn volwassen leden (18 jaar of ouder) van het huishouden?**

________________________________________________________________

Q343 [IWER: Noteer het aantal niet-volwassen leden van het huishouden] $e{ q://QID86/ChoiceTextEntryValue - q://QID342/ChoiceTextEntryValue }

________________________________________________________________

1. SOCIO-DEMOGRAPHIC INFORMATION

Q154 [INLEIDING]

**De volgende reeks vragen die ik u zal stellen hebben betrekking op uw PERSOONLIJKE situatie. We zullen starten met enkele vragen over uw nationaliteit, opleiding en eventuele huidige tewerkstelling.**

Q161 **Wat is het hoogste opleidingsniveau dat u heeft behaald?**
[IWER: Spontaan antwoord. Selecteer de juiste antwoordcategorie en bevestig deze luidop]
[IWER: maar één antwoord mogelijk.]

- 1. Basisonderwijs (1)
- 2. Lager secudair onderwijs (inclusief algemeen onderwijs, kunstonderwijs, technisch onderwijs en beroepsonderwijs) (2)
- 3. Hoger secundair onderwijs (inclusief algemeen onderwijs, kunstonderwijs, technisch onderwijs en beroepsonderwijs) (3)
- 4. Niet-universitair hoger onderwijs korte cyclus (2 of 3 jaar) (4)
- 5. Niet-universitair hoger onderwijs lange cyclus (4 of 5 jaar) (5)
- 6. Universitair onderwijs (6)
- 7. Geen (7)
- 8. Andere, maar niet erkend in België (8)
- 9. Andere, specifieer: (9) ________________________________________________
- Ik weet het niet (10)
- Weigering (11)

Q162 **Gelieve antwoordkaart 2 te bekijken. Welke van de volgende categorieën beschrijft uw huidige activiteitsstatus het best?**
[IWER: maar één antwoord mogelijk.]

- 1. Loontrekkende (1)
- 2. Zelfstandige in hoofdberoep (2)
- 3. Werkloos (3)
- 4. Tewerkstellingsprogramma (bijvoorbeeld artikel 60) (4)
- 5. In (beroeps) opleiding / omscholing (5)
- 6. In ouderschapsverlof (6)
- 7. Op pensioen of vervoegd pensioen (7)
- 8. Op brugpensioen (8)
- 9. Invalide of arbeidsongeschikt (9)
- 10. Huisman/vrouw (10)
- 11. Loopbaanonderbreking of in tijdskrediet maar geregistreerd bij RVA (11)
- 12. Andere, specifieer: (12) ________________________________________________
- Ik weet het niet (13)
- Weigering (14)

1. HOUSEHOLD INCOME

Q135 **De volgende vragen hebben betrekking op het totale inkomen van UW HUISHOUDEN. Het totaal beschikbaar inkomen van een huishouden bestaat uit: (1) netto-inkomens uit werk;** **(2) sociale uitkeringen bijvoordbeeld het leefloon, kinderbijslagen, enz.; (3) bijkomende inkomens zoals huuropbrengsten, e.a. en (4) vergoedingen om klusjes uit te voeren, of van bijverdiensten** **van ALLE personen uit uw huishouden SAMEN (al dan niet aangegeven).**     **In het geval u zich in een regeling van collectieve schuldbemiddeling bevindt, gelieve het bedrag dat maandelijks naar de afbetaling van uw schulden gaat hier toch bij te tellen.**
**In het geval u maandelijks alimentatie dient te betalen, gelieve het bedrag dat u hiervoor maandelijks betaald nog wel bij u totale huishoudinkomen te tellen. In het geval u maandelijks alimentatie ontvangt, gelieve dit bedrag hier nog niet bij te tellen.**

Q136 **Hoeveel bedroeg het totaal beschikbaar HUISHOUD inkomen vorige maand? Als dit inkomen varieert van maand tot maand, gelieve dan een gemiddelde te geven.**

- Bedrag per maand: (1) ________________________________________________
- Ik weet het niet (2)
- Weigering (3)

Display This Question:

If Hoeveel bedroeg het totaal beschikbaar HUISHOUD inkomen vorige maand? Als dit inkomen varieert van maand tot maand, gelieve dan een gemiddelde te geven. = Ik weet het niet

Or Hoeveel bedroeg het totaal beschikbaar HUISHOUD inkomen vorige maand? Als dit inkomen varieert van maand tot maand, gelieve dan een gemiddelde te geven. = Weigering

Q137 **Gelieve antwoordkaart 10 te bekijken. Kunt u me vertellen in welke categorie het maandelijks totaal beschikbaar inkomen van uw huishouden (inclusief lonen, uitkeringen, huuropbrengsten, bijverdiensten, etc.) zich vorige maand bevond?**

- 1. Minder dan 249€ per maand (1)
- 2. Tussen 250 en 499€ per maand (2)
- 3. Tussen 500 en 749€ per maand (3)
- 4. Tussen 750 en 999€ per maand (4)
- 5. Tussen 1000 en 1499€ per maand (5)
- 6. Tussen 1500 en 1999€ per maand (6)
- 7. Tussen 2000 en 2499€ per maand (7)
- 8. Tussen 2500 en 2999€ per maand (8)
- 9. Tussen 3000 en 4999€ per maand (9)
- 10. 5000€ of meer per maand (10)
- Ik weet het niet (11)
- Weigering (12)

Q140 **Met het totaal beschikbaar inkomen van uw huishouden voor ogen, is uw huishouden in staat om maandelijks rond komen, met ander woorden de gebruikelijke uitgaven te betalen?**
[IWER: lees volgende opties luidop voor]

- 1. Zeer moeilijk (73)
- 2. Moeilijk (74)
- 3. Eerder moeilijk (75)
- 4. Eerder gemakkelijk (76)
- 5. Gemakkelijk (77)
- 6. Zeer gemakkelijk (78)
- Ik weet het niet (79)
- Weigering (80)

1. SOCIAL ASSISTANCE FOR PERSONS AT ACTIVE AGE

Q229 **Kent u het leefloon?**

- Ja (1)
- Nee (2)
- Ik weet het niet (3)
- Weigering (4)

Display This Question:

If Kent u het leefloon?  = Nee

Or Kent u het leefloon?  = Ik weet het niet

Or Kent u het leefloon?  = Weigering

Q230 [IWER: lees de volgende beschrijving voor].

**Leefloon = Het leefloon is een vorm van financiële hulp die wordt toegekend aan mensen die het moeilijk hebben om rond te komen. Het leefloon maakt deel uit van het recht op maatschappelijke integratie, wat vroeger het bestaansminimum werd genoemd. Om het leefloon te ontvangen moet je voldoen aan een aantal voorwaarden en een aanvraag indienen bij het OCMW kantoor in uw buurt.**   **Herkent u de uitkering van de beschrijving die u net heeft gehoord?**

- Ja (1)
- Nee (2)
- Ik weet het niet (3)
- Weigering (4)

Skip To: End of Block If [IWER: lees de volgende beschrijving voor].   Leefloon = Het leefloon is een vorm van financiële hulp die wordt toegekend aan mensen die het moeilijk hebben om rond te komen. Het leefloon maakt deel uit van het recht op maatschappelijke integratie, wat vroeger het bestaansminimum werd genoemd. Om het leefloon te ontvangen moet je voldoen aan een aantal voorwaarden en een aanvraag indienen bij het OCMW kantoor in uw buurt.   Herkent u de uitkering van de beschrijving die u net heeft gehoord? = Nee

Skip To: End of Block If [IWER: lees de volgende beschrijving voor].   Leefloon = Het leefloon is een vorm van financiële hulp die wordt toegekend aan mensen die het moeilijk hebben om rond te komen. Het leefloon maakt deel uit van het recht op maatschappelijke integratie, wat vroeger het bestaansminimum werd genoemd. Om het leefloon te ontvangen moet je voldoen aan een aantal voorwaarden en een aanvraag indienen bij het OCMW kantoor in uw buurt.   Herkent u de uitkering van de beschrijving die u net heeft gehoord? = Ik weet het niet

Skip To: End of Block If [IWER: lees de volgende beschrijving voor].   Leefloon = Het leefloon is een vorm van financiële hulp die wordt toegekend aan mensen die het moeilijk hebben om rond te komen. Het leefloon maakt deel uit van het recht op maatschappelijke integratie, wat vroeger het bestaansminimum werd genoemd. Om het leefloon te ontvangen moet je voldoen aan een aantal voorwaarden en een aanvraag indienen bij het OCMW kantoor in uw buurt.   Herkent u de uitkering van de beschrijving die u net heeft gehoord? = Weigering

Q244
**Gelieve antwoordkaart 15 te bekijken. Ik zal u een aantal stellingen voorlezen omtrent uw KENNIS over het aanvragen van een leefloon. Voor elke stelling, zou u me kunnen zeggen of u akkoord bent of niet?**

 **Ik ken de voordelen van het leefloon**

- 1. Helemaal akkoord (1)
- 2. Akkoord (2)
- 3. Noch akkoord, noch niet akkoord (3)
- 4. Niet akkoord (4)
- 5. Helemaal niet akkoord (5)
- Ik weet het niet (6)
- Weigering (7)

Q444 **(Gelieve naar antwoordkaart 15 te kijken) Ik heb een redelijk goed idee of ik in aanmerking kom voor een leefloon**

- 1. Helemaal akkoord (1)
- 2. Akkoord (2)
- 3. Noch akkoord, noch niet akkoord (3)
- 4. Niet akkoord (4)
- 5. Helemaal niet akkoord (5)
- Ik weet het niet (6)
- Weigering (7)

Q445 **(Gelieve naar antwoordkaart 15 te kijken) Ik ken de procedure voor het aanvragen van een leefloon**

- 1. Helemaal akkoord (1)
- 2. Akkoord (2)
- 3. Noch akkoord, noch niet akkoord (3)
- 4. Niet akkoord (4)
- 5. Helemaal niet akkoord (5)
- Ik weet het niet (6)
- Weigering (7)

Q245 **(Gelieve naar antwoordkaart 15 te kijken) Ik zal u een aantal stellingen voorlezen over de AANVRAAGPROCEDURE voor het leefloon. Voor elke stelling, zou u me kunnen zeggen of u akkoord bent of niet?**

**Het is veel werk om het leefloon aan te vragen**

- 1. Helemaal akkoord (1)
- 2. Akkoord (2)
- 3. Noch akkoord, noch niet akkoord (3)
- 4. Niet akkoord (4)
- 5. Helemaal niet akkoord (5)
- Ik weet het niet (6)
- Weigering (7)

Q570 **(Gelieve naar antwoordkaart 15 te kijken) De procedure om het leefloon aan te vragen is moeilijk**

- 1. Helemaal akkoord (1)
- 2. Akkoord (2)
- 3. Noch akkoord, noch niet akkoord (3)
- 4. Niet akkoord (4)
- 5. Helemaal niet akkoord (5)
- Ik weet het niet (6)
- Weigering (7)

Q567 **(Gelieve naar antwoordkaart 15 te kijken) Alles bij elkaar genomen, duurt het lang om het leefloon aan te vragen**

- 1. Helemaal akkoord (1)
- 2. Akkoord (2)
- 3. Noch akkoord, noch niet akkoord (3)
- 4. Niet akkoord (4)
- 5. Helemaal niet akkoord (5)
- Ik weet het niet (6)
- Weigering (7)

Q460 **Gelieve antwoordkaart 15 te bekijken. Ik zal u een aantal stellingen voorlezen over het leefloon. Voor elke stelling, zou u me kunnen zeggen of u akkoord bent of niet?**

**Als iemand het leefloon krijgt, zou die zich daar voor moeten schamen**

- 1. Helemaal akkoord (1)
- 2. Akkoord (2)
- 3. Noch akkoord, noch niet akkoord (3)
- 4. Niet akkoord (4)
- 5. Helemaal niet akkoord (5)
- Ik weet het niet (6)
- Weigering (7)

Q459 **(Gelieve naar antwoordkaart 15 te kijken) De mensen met wie je omgaat, kijken op je neer wanneer je een leefloon ontvangt**

- 1. Helemaal akkoord (1)
- 2. Akkoord (2)
- 3. Noch akkoord, noch niet akkoord (3)
- 4. Niet akkoord (4)
- 5. Helemaal niet akkoord (5)
- Ik weet het niet (6)
- Weigering (7)

Q456 **(Gelieve naar antwoordkaart 15 te kijken) Het aanvragen van een leefloon, zou me het gevoel geven te bedelen**

- 1. Helemaal akkoord (1)
- 2. Akkoord (2)
- 3. Noch akkoord, noch niet akkoord (3)
- 4. Niet akkoord (4)
- 5. Helemaal niet akkoord (5)
- Ik weet het niet (6)
- Weigering (7)

Q461 **(Gelieve naar antwoordkaart 15 te kijken) Als ik een leefloon zou krijgen, dan zou ik me daar voor schamen**

- 1. Helemaal akkoord (1)
- 2. Akkoord (2)
- 3. Noch akkoord, noch niet akkoord (3)
- 4. Niet akkoord (4)
- 5. Helemaal niet akkoord (5)
- Ik weet het niet (6)
- Weigering (7)

*****FRENCH VERSION*****

1. INTRODUCTION, INFORMED CONSENT AND SELECTION MAIN RESPONDENT

Q752 Veuillez encoder l'identifiant du ménage:

________________________________________________________________

Q836 [ENQUÊTEUR: Veuillez sélectionner la Région dans laquelle vit le ménage.]

- Bruxelles (1)
- Flandre (2)
- Wallonie (3)

QID80 CE MODULE EST UNIQUEMENT DESTINÉ À LA PERSONNE DE RÉFÉRENCE. VEUILLEZ LA SELECTIONNER ATTENTIVEMENT.

QID81 [Introduction générale pour l'enquêteur.]

**Par cette étude, nous souhaitons mesurer l'accessibilité des aides et des services en Belgique ainsi que le nombre de personnes qui en bénéficient. Plus particulièrement, nous voulons savoir s'il y a beaucoup de personnes qui ont droit à une aide mais qui ne la reçoivent pas. Dans ce but, je vais vous poser des questions sur votre composition familiale, votre logement, votre éventuel travail, votre expérience avec les procédures de demande, pourquoi par le passé vous n'avez pas demandé certaines aides, etc. . Je vais aussi vous poser des questions financières. Ces questions sont nécessaires afin de déterminer si quelqu'un a droit ou non à une aide sociale. Si vous souhaitez ne pas répondre à une question, dites-le moi et je passerai à la suivante. Nous vous garantissons que toutes vos réponses seront traitées de manière anonyme et confidentielle. Votre participation est entièrement volontaire et que vous répondiez ou non à ces questions, vos droits sociaux ne seront pas modifiés suite à cette étude.**

Q754 [Introduction de l'enquêteur concernant le lien aux données administratives.]

**Pour que l'entretien soit aussi court que possible, nous voudrions lier vos réponses avec certaines données déjà existantes dans les bases de données administratives. Cela concerne le droit à l’aide sociale et certaines conditions spécifiques pour l’obtenir. Afin de ne pas vous poser des questions sur ces sujets, je voudrais vous demander votre accord pour que les chercheurs puissent utiliser les données administratives déjà existantes.

Ces données et vos réponses au questionnaire ne seront utilisées que par les chercheurs. Afin de respecter strictement la législation sur la protection de la vie privée, toutes les données seront cryptées. Cela veut dire que vos nom et adresse resteront anonymes. De plus, les données collectées ne seront utilisées que pour réaliser des travaux statistiques, en aucun cas dans un but administratif.

Enfin, je voudrais à nouveau mettre en avant le fait que votre participation et votre accord à ce que vos données administratives soient utilisées n'auront aucune conséquence négative et n'affecteront aucunement vos droits actuels.**
 
**Acceptez-vous que nous lions vos réponses aux données administratives?**

- Oui (1)
- Non [ENQUÊTEUR: le questionnaire prend fin.] (2)

Skip To: End of Survey If [Introduction de l'enquêteur concernant le lien aux données administratives.]Pour que l'entretien soit aussi court que possible, nous voudrions lier vos réponses avec certaines données déjà existantes dans les bases de données administratives. Cela concerne le droit à l’aide sociale et certaines conditions spécifiques pour l’obtenir. Afin de ne pas vous poser des questions sur ces sujets, je voudrais vous demander votre accord pour que les chercheurs puissent utiliser les données administratives déjà existantes. Ces données et vos réponses au questionnaire ne seront utilisées que par les chercheurs. Afin de respecter strictement la législation sur la protection de la vie privée, toutes les données seront cryptées. Cela veut dire que vos nom et adresse resteront anonymes. De plus, les données collectées ne seront utilisées que pour réaliser des travaux statistiques, en aucun cas dans un but administratif. Enfin, je voudrais à nouveau mettre en avant le fait que votre participation et votre accord à ce que vos données administratives soient utilisées n'auront aucune conséquence négative et n'affecteront aucunement vos droits actuels.   Acceptez-vous que nous lions vos réponses aux données administratives? = Non [ENQUÊTEUR: le questionnaire prend fin.]

QID82 **Au sein de votre ménage, êtes-vous la personne principale qui réalise les demandes pour les aides sociales éventuelles et qui a une bonne connaissance des finances du ménage? Si vous n'êtes pas cette personne, pouvez-vous alors m'indiquer qui est la personne de référence?**

- Oui, le/la répondant(e) est bien placé(e) pour répondre au questionnaire (1)
- Non, quelqu'un d'autre dans le ménage est mieux placé pour répondre à ce questionnaire. [ENQUÊTEUR: réponse orale, le questionnaire prend fin.] (2)

Skip To: End of Survey If Au sein de votre ménage, êtes-vous la personne principale qui réalise les demandes pour les aides sociales éventuelles et qui a une bonne connaissance des finances du ménage? Si vous n'êtes pas cette personne, pouvez-vous alors m'indiquer qui est la personne de référence? = Non, quelqu'un d'autre dans le ménage est mieux placé pour répondre à ce questionnaire. [ENQUÊTEUR: réponse orale, le questionnaire prend fin.]

Q849 [ENQUÊTEUR: notez le sexe du répondant selon votre observation. Ne demandez qu'en cas de doute]

- Homme (1)
- Femme (2)
- Transgenre (3)

**Quel âge avez-vous?**

[Encodez 200 si le réspondant ne sait pas.]

________________________________________________________________

1. HOUSEHOLD COMPOSITION

QID85 **Commençons par la composition de votre ménage et les relations entre vous et les personnes mentionnées.**

QID86 **Actuellement, SANS VOUS COMPTER mais enfants compris, combien de personnes vivent ici régulièrement en tant que membres du ménage? Un ménage est constitué de toutes les personnes vivant à la MÊME ADRESSE (même porte d'entrée), ayant un BUDGET COMMUN ou MANGEANT GÉNÉRALEMENT ENSEMBLE.**

[ENQUÊTEUR: Les personnes suivantes peuvent être considérées comme des membres du ménage: les enfants vivant avec leurs parents à la même adresse, ou des parents vivant avec leurs enfants à l'adresse de ces derniers; les enfants vivant à temps partiel avec le répondant, même s'ils sont domiciliés chez l’ex partenaire (ou ailleurs) mais qui sont financièrement dépendants du répondant; les personnes officiellement enregistrées comme vivant à cette adresse et dans le cas de dépendance financière, mais qui temporairement travaillent ou étudient trop loin pour revenir chaque jour (par exemple les étudiants vivant en kots); les personnes résidant temporairement à l'hôpital, à l'étranger ou autre part, si le répondant considère qu'ils sont des membres du ménage.
Ne sont généralement pas considérés comme membres du ménage: les colocataires (location d'une chambre), même lorsqu'ils mangent parfois avec le répondant, à moins que le répondant ne les mentionne expressément comme membres du ménage.]

________________________________________________________________

Skip To: End of Block If Condition: Actuellement, SANS VOUS COMPTER mais enfants compris, combien de personnes vivent ici régulièrement en tant que membres du ménage? Un ménage est constitué de toutes les personnes vivant à la MÊME ADRESSE (même porte d'entrée), ayant un BUDGET COMMUN ou MANGEANT GÉNÉRALEMENT ENSEMBLE. [ENQUÊTEUR: Les personnes suivantes peuvent être considérées comme des membres du ménage: les enfants vivant avec leurs parents à la même adresse, ou des parents vi ... Is Equal to 0. Skip To: End of Block.

Q341 **TOUJOURS SANS VOUS COMPTER, combien d'entre eux sont majeurs (18 ans ou plus)?**

________________________________________________________________

Q343 [Enquêteur: encodez simplement le nombre qui apparaît, relatif aux personnes mineures (- de 18 ans).]: $e{ q://QID86/ChoiceTextEntryValue - q://QID342/ChoiceTextEntryValue }

________________________________________________________________

1. SOCIO-DEMOGRAPHIC INFORMATION

Q154   **La prochaine série de questions concerne votre situation PERSONNELLE. Je voudrais commencer cette partie par des questions sur votre nationalité, votre formation et votre statut professionnel.**

Q161 **Quel est le plus haut niveau de diplôme que vous ayez obtenu?**

[ENQUÊTEUR: réponse spontanée. Encodez l'option de réponse correspondante et confirmez oralement.]
[ENQUÊTEUR: Une seule réponse possible.]

- 1. Certificat d'études primaires (CEB) (1)
- 2. Enseignement secondaire inférieur (y compris l’enseignement général, artistique, technique ou professionnel) (2)
- 3. Enseignement secondaire supérieur (y compris l’enseignement général, artistique, technique ou professionnel) (3)
- 4. Enseignement supérieur non-universitaire de type court (2 ou 3 ans) (4)
- 5. Enseignement supérieur non-universitaire de type long (4 ou 5 ans) (5)
- 6. Diplôme universitaire (6)
- 7. Aucun (7)
- 8. Autre diplôme mais pas reconnu en Belgique (8)
- 9. Autre, précisez: (9) ________________________________________________
- Je ne sais pas (10)
- Refus (11)

Q162 **Veuillez consulter la fiche 2. Quel statut décrit le mieux votre situation professionnelle actuelle principale?**
[ENQUÊTEUR: Une seule réponse possible.]

- 1. Employé ou salarié (1)
- 2. Indépendant (2)
- 3. Sans emploi (3)
- 4. Programme d'insertion professionnelle (par exemple: Article 60) (4)
- 5. En formation professionnelle ou recyclage (5)
- 6. En congé parental (6)
- 7. Retraité ou pré-retraité (7)
- 8. Chômage avec complément d'entreprise (pour les travailleurs d'un certain âge ayant été licenciés) (8)
- 9. Invalide ou en incapacité de travail (9)
- 10. Au foyer (10)
- 11. Interruption de carrière ou crédit temps, déclaré à l'ONEM (11)
- 12. Autre, précisez: (12) ________________________________________________
- Je ne sais pas (13)
- Refus (14)

Start of Block: Household income and financial situation

1. HOUSEHOLD INCOME

Q135 **Les questions suivantes concernent le revenu total de VOTRE MÉNAGE. Le revenu total disponible d’un ménage se compose :** **(1) des salaires nets issus du travail;** **(2) des aides sociales, par exemple le revenu d'intégration du CPAS, les allocations familiales, etc.;** **(3) des revenus complémentaires comme des revenus de location, etc.;** **(4) des revenus de petits travaux ou honoraires / pourboires de TOUTES les personnes du ménage pris ENSEMBLE, même non déclarés.**   **Si vous êtes en médiation de dettes, veuillez COMPTER dans le calcul du revenu total de votre ménage le montant qui vous est prélevé chaque mois pour le remboursement de vos dettes.**
**Si vous payez une pension alimentaire, afin de calculer le revenu total de votre ménage, veuillez prendre en compte la pension que vous versez mensuellement. Si vous recevez une pension alimentaire, veuillez ne pas la prendre en compte (pour le moment) dans le montant total que vous recevez mensuellement.**

Q136 **Quel était le revenu mensuel total disponible DE VOTRE MÉNAGE le mois dernier ? Si ces revenus varient d’un mois à l’autre, veuillez donner une moyenne.**

- Montant par mois: (1) ________________________________________________
- Je ne sais pas (2)
- Refus (3)

Display This Question:

If Quel était le revenu mensuel total disponible DE VOTRE MÉNAGE le mois dernier ? Si ces revenus varient d’un mois à l’autre, veuillez donner une moyenne. = Je ne sais pas

Or Quel était le revenu mensuel total disponible DE VOTRE MÉNAGE le mois dernier ? Si ces revenus varient d’un mois à l’autre, veuillez donner une moyenne. = Refus

Q137 **Merci de regarder la fiche 10 et de m’indiquer la fourchette du montant approximatif que représente le revenu moyen mensuel (salaires, prestations sociales, recettes de location, pourboires,… compris) total disponible de VOTRE MÉNAGE le mois dernier?**

- 1. Moins de 249€ par mois (1)
- 2. Entre 250 et 499€ par mois (2)
- 3. Entre 500 et 749€ par mois (3)
- 4. Entre 750€ et 999€ par mois (4)
- 5. Entre 1000 et 1499€ par mois (5)
- 6. Entre 1500 et 1999€ par mois (6)
- 7. Entre 2000 et 2499€ par mois (7)
- 8. Entre 2500 et 2999€ par mois (8)
- 9. Entre 3000 et 4999€ par mois (9)
- 10. 5000€ ou plus par mois (10)
- Je ne sais pas (11)
- Refus (12)

Q140 **Pensez aux revenus totaux de votre ménage. Êtes-vous en mesure de joindre les deux bouts chaque mois, c’est-à-dire de payer vos dépenses habituelles?**

 [ENQUÊTEUR: Lisez à voix haute les options de réponses suivantes.]

- 1. Très difficilement (73)
- 2. Difficilement (74)
- 3. Plutôt difficilement (75)
- 4. Plutôt facilement (76)
- 5. Facilement (77)
- 6. Très facilement (78)
- Je ne sais pas (79)
- Refus (80)

1. SOCIAL ASSISTANCE FOR PERSONS AT ACTIVE AGE

Q229 **Connaissez-vous le revenu d'intégration du CPAS?**

- Oui (1)
- Non (2)
- Je ne sais pas (3)
- Refus (4)

Display This Question:

If Connaissez-vous le revenu d'intégration du CPAS?  = Non

Or Connaissez-vous le revenu d'intégration du CPAS?  = Je ne sais pas

Or Connaissez-vous le revenu d'intégration du CPAS?  = Refus

Q230 [ENQUÊTEUR: lisez la description suivante.]

**Le revenu d'intégration est une forme d'aide accordée aux personnes difficultés financières. Le revenu d'intégration fait partie du droit à l'intégration sociale, qui était auparavant appelé le Minimex (Minimum des Moyens d'Existence). Pour obtenir le revenu d'intégration, vous devez remplir un certain nombre de critères et effectuer une demande auprès du CPAS de votre commune.**   **Reconnaissez-vous l'allocation sur base de la description ?**

- Oui (1)
- Non (2)
- Je ne sais pas (3)
- Refus (4)

Skip To: End of Block If [ENQUÊTEUR: lisez la description suivante.]   Le revenu d'intégration est une forme d'aide accordée aux personnes difficultés financières. Le revenu d'intégration fait partie du droit à l'intégration sociale, qui était auparavant appelé le Minimex (Minimum des Moyens d'Existence). Pour obtenir le revenu d'intégration, vous devez remplir un certain nombre de critères et effectuer une demande auprès du CPAS de votre commune.   Reconnaissez-vous l'allocation sur base de la description ? = Non

Skip To: End of Block If [ENQUÊTEUR: lisez la description suivante.]   Le revenu d'intégration est une forme d'aide accordée aux personnes difficultés financières. Le revenu d'intégration fait partie du droit à l'intégration sociale, qui était auparavant appelé le Minimex (Minimum des Moyens d'Existence). Pour obtenir le revenu d'intégration, vous devez remplir un certain nombre de critères et effectuer une demande auprès du CPAS de votre commune.   Reconnaissez-vous l'allocation sur base de la description ? = Je ne sais pas

Skip To: End of Block If [ENQUÊTEUR: lisez la description suivante.]   Le revenu d'intégration est une forme d'aide accordée aux personnes difficultés financières. Le revenu d'intégration fait partie du droit à l'intégration sociale, qui était auparavant appelé le Minimex (Minimum des Moyens d'Existence). Pour obtenir le revenu d'intégration, vous devez remplir un certain nombre de critères et effectuer une demande auprès du CPAS de votre commune.   Reconnaissez-vous l'allocation sur base de la description ? = Refus

Q244 **Veuillez consulter la fiche 15. Je vais vous lire quelques énoncés à propos de vos CONNAISSANCES sur la procédure de demande pour le revenu d’intégration. Pour chacun, pouvez-vous me dire si vous êtes d’accord ou pas ?**

 **Je connais les avantages du revenu d'intégration.**

- 1. Tout à fait d'accord (1)
- 2. D'accord (2)
- 3. Ni d'accord, ni pas d'accord (3)
- 4. Pas d'accord (4)
- 5. Pas du tout d'accord (5)
- Je ne sais pas (6)
- Refus (7)

Q444 **(Veuillez consulter la fiche 15) Je sais assez bien si j'ai droit au revenu d'intégration.**

- 1. Tout à fait d'accord (1)
- 2. D'accord (2)
- 3. Ni d'accord, ni pas d'accord (3)
- 4. Pas d'accord (4)
- 5. Pas du tout d'accord (5)
- Je ne sais pas (6)
- Refus (7)

Q445 **(Veuillez consulter la fiche 15) Je connais la procédure de demande pour le revenu d'intégration.**

- 1. Tout à fait d'accord (1)
- 2. D'accord (2)
- 3. Ni d'accord, ni pas d'accord (3)
- 4. Pas d'accord (4)
- 5. Pas du tout d'accord (5)
- Je ne sais pas (6)
- Refus (7)

Q245 **(Veuillez consulter la fiche 15) Je vais vous lire quelques énoncés à propos de la PROCÉDURE DE DEMANDE POUR le revenu d’intégration. Pour chacun, pouvez-vous me dire si vous êtes d’accord ou non.**

**Cela demande beaucoup de travail de demander le revenu d'intégration**

- 1. Tout à fait d'accord (1)
- 2. D'accord (2)
- 3. Ni d'accord, ni pas d'accord (3)
- 4. Pas d'accord (4)
- 5. Pas du tout d'accord (5)
- Je ne sais pas (6)
- Refus (7)

Q570 **(Veuillez consulter la fiche 15) La procédure de demande du revenu d'intégration est difficile.**

- 1. Tout à fait d'accord (1)
- 2. D'accord (2)
- 3. Ni d'accord, ni pas d'accord (3)
- 4. Pas d'accord (4)
- 5. Pas du tout d'accord (5)
- Je ne sais pas (6)
- Refus (7)

Q567 **(Veuillez consulter la fiche 15) En tenant compte de tout, la demande de revenu d'intégration prend beaucoup de temps.**

- 1. Tout à fait d'accord (1)
- 2. D'accord (2)
- 3. Ni d'accord, ni pas d'accord (3)
- 4. Pas d'accord (4)
- 5. Pas du tout d'accord (5)
- Je ne sais pas (6)
- Refus (7)

Q460

**Veuillez consulter la fiche 15. Je vais vous lire quelques énoncés à propos du revenu d’intégration. Pour chacun, pouvez-vous me dire si vous êtes d’accord ou pas ?**

**Si quelqu’un reçoit le revenu d’intégration, il doit avoir honte.**

- 1. Tout à fait d'accord (1)
- 2. D'accord (2)
- 3. Ni d'accord, ni pas d'accord (3)
- 4. Pas d'accord (4)
- 5. Pas du tout d'accord (5)
- Je ne sais pas (6)
- Refus (7)

Q459 **(Veuillez consulter la fiche 15) Les personnes que vous fréquentez vous méprisent parce que vous recevez le revenu d’intégration.**

- 1. Tout à fait d'accord (1)
- 2. D'accord (2)
- 3. Ni d'accord, ni pas d'accord (3)
- 4. Pas d'accord (4)
- 5. Pas du tout d'accord (5)
- Je ne sais pas (6)
- Refus (7)

Q456 **(Veuillez consulter la fiche 15) Demander le revenu d'intégration me donnerait l'impression de mendier.**

- 1. Tout à fait d'accord (1)
- 2. D'accord (2)
- 3. Ni d'accord, ni pas d'accord (3)
- 4. Pas d'accord (4)
- 5. Pas du tout d'accord (5)
- Je ne sais pas (6)
- Refus (7)

Q461 **(Veuillez consulter la fiche 15) Si je recevais le revenu d'intégration, j'aurais honte**

- 1. Tout à fait d'accord (1)
- 2. D'accord (2)
- 3. Ni d'accord, ni pas d'accord (3)
- 4. Pas d'accord (4)
- 5. Pas du tout d'accord (5)
- Je ne sais pas (6)
- Refus (7)
